# Do Patients Taking Warfarin Experience Delays to Theatre, Longer Hospital Stay, and Poorer Survival After Hip Fracture?

**DOI:** 10.1007/s11999-016-5056-0

**Published:** 2016-09-01

**Authors:** John E. Lawrence, Daniel M. Fountain, Duncan J. Cundall-Curry, Andrew D. Carrothers

**Affiliations:** 1 Department of Trauma and Orthopaedics, Addenbrooke’s Hospital, Hills Road, Cambridge, CB2 0QQ UK; 2 School of Clinical Medicine, University of Cambridge, Cambridge, UK

## Abstract

**Background:**

Patients sustaining a fractured neck of the femur are typically of advanced age with multiple comorbidities. As a consequence, the proportion of these patients receiving warfarin therapy is approximately 10%. There are currently few studies investigating outcomes in this subset of patients.

**Questions/purposes:**

The purpose of this study was to assess the association between warfarin therapy and time to surgery, length of hospital stay, and survival in patients sustaining a fractured neck of the femur.

**Methods:**

Data for 2036 patients admitted to our center between July 2009 and July 2014 with a fractured neck of the femur were extracted from the National Hip Fracture Database. Fifty-seven patients received no surgical treatment and were excluded from analysis. Multivariable ordinary least squares regression was performed to test the association between warfarin treatment on time to surgery and length of stay, and Cox proportional hazards to test followup survival. Variables included in the regression model were age, sex, American Society of Anesthesiologists (ASA) score, admission Abbreviated Mental Test Score (AMTS), fracture type, operation type, and premorbid Work Ability Index (WAI). One hundred fifty-two of 1979 surgically treated patients (8%) were receiving warfarin therapy at the time of admission.

**Results:**

After controlling for age, sex, ASA score, AMTS, fracture type, operation type, and WAI, we found that patients taking warfarin were less likely to go to surgery by 36 hours (odds ratio [OR], 0.20; 95% CI, 0.14–0.30), and less likely to go to surgery by 48 hours (OR, 0.17; 95% CI, 0.11–0.24). Patients taking warfarin had a longer length of stay (median, 15 days; interquartile range [IQR], 12–22 days) compared with patients not taking warfarin (median, 13 days; IQR, 9–20 days; p < 0.001). Survival analysis to June 2015 showed a higher mortality for patients taking warfarin (12-month survival, 66% vs 76%; hazard ratio, 1.57; 95% CI, 1.21–2.04; p < 0.001).

**Conclusions:**

After controlling for multiple prognostic factors such as age, ASA score, AMTS, and WAI, warfarin therapy at the time of injury is associated with increased time to surgery, length of stay, and decreased survival. This study highlights the need to view warfarin therapy as a ‘red flag’ in patients presenting with a fractured neck of the femur. Preoperatively, prompt warfarin reversal together with adequate investigation and optimization of the patient should ensure timely, safe surgery. Early involvement of the anesthesia team should ensure an appropriate level of postoperative care for these patients.

**Level of Evidence:**

Level III, therapeutic study

**Electronic supplementary material:**

The online version of this article (doi:10.1007/s11999-016-5056-0) contains supplementary material, which is available to authorized users.

## Introduction

Hip fractures account for the majority of orthopaedic trauma in the United Kingdom (UK), with more than 64,000 cases in England, Wales, and Northern Ireland in 2014 [20]. The condition often affects elderly patients with multiple comorbidities and consequently has been shown to have poor clinical outcomes, with mortality at 12 months as much as 36% [1, 4]. In the United States, at least 250,000 people are hospitalized with hip fractures annually [6]. This global public health problem is expected to worsen, with sharp increases in the number of hip fractures forecast for the next generation [18]. Despite focus on improving the care of these patients, postinjury mortality remains stubbornly high [11].

Patients who sustain fragility fractures of the hip often have concurrent comorbidities, some of which require anticoagulation with warfarin. Between 7.8% and 10.3% of all patients admitted with these fractures are receiving long-term warfarin therapy, which produces a coagulopathic state as expressed by increased International Normalized Ratio (INR) [8, 9, 25]. This coagulopathy must be reduced or eliminated before surgery can safely be performed. The time taken to achieve a safe INR together with the presence of multiple comorbidities has the potential to delay surgical treatment, which conflicts with policy initiatives (such as the 2010 ‘Best Practice Tariff’ in the UK) that aim to improve patient outcomes by reducing delays in hip fracture surgery to less than 48 or 36 hours [5, 19]. Few studies have investigated the degree to which warfarin therapy is associated with delays to surgery [2, 7, 8, 16], and the degree to which such warfarin-associated care delays are associated with length of stay and mortality has not been reported, to our knowledge.

We sought to determine whether a patient’s use of warfarin at the time of admission for a femoral neck fracture was associated with (1) time to hip-fracture surgery, (2) length of hospital stay, and (3) survival in patients sustaining a fractured neck of the femur.

## Patients and Methods

**Fig. 1:**
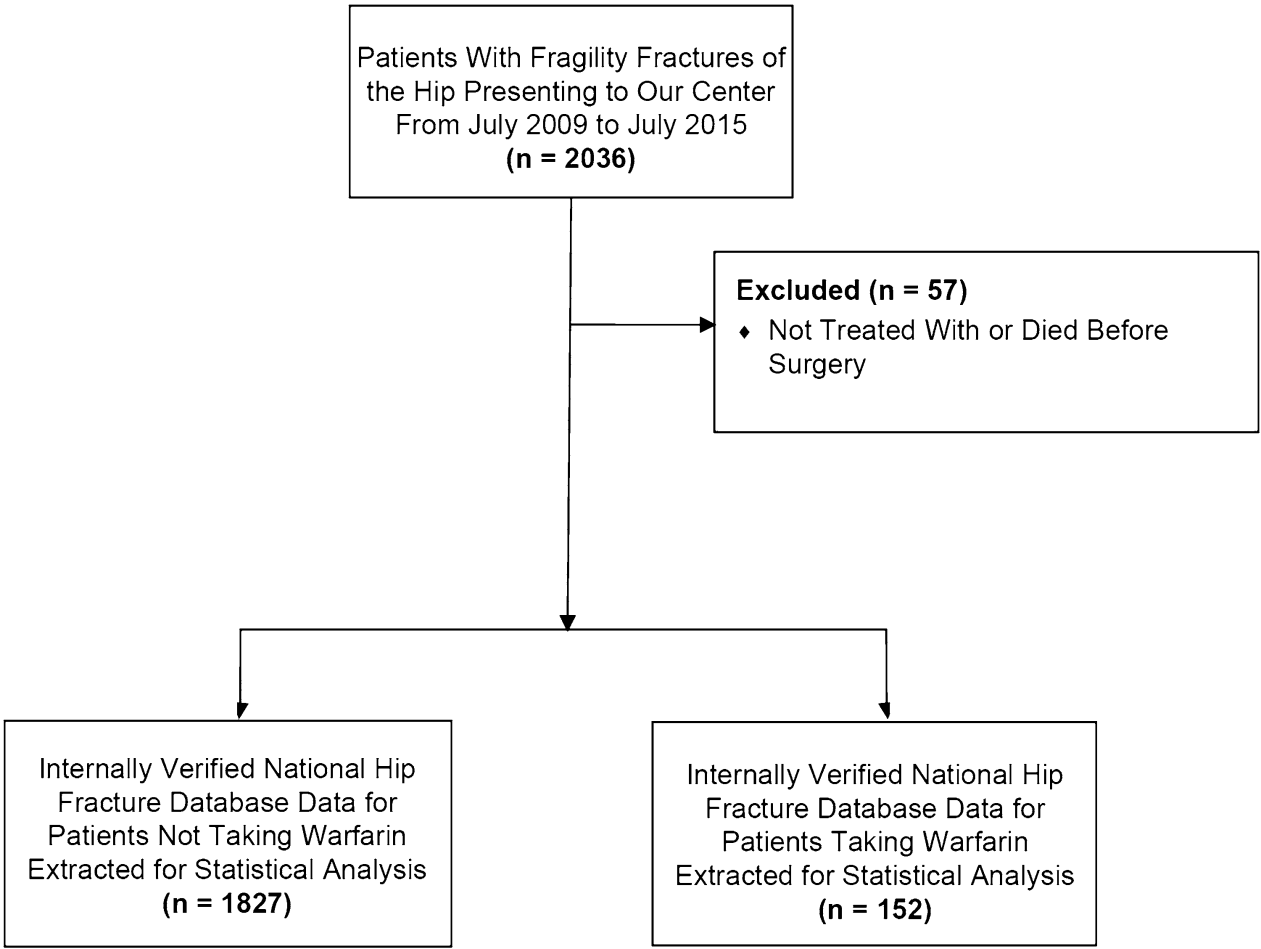
The flow diagram shows the selection of patient data for our study.

**Fig. 2:**
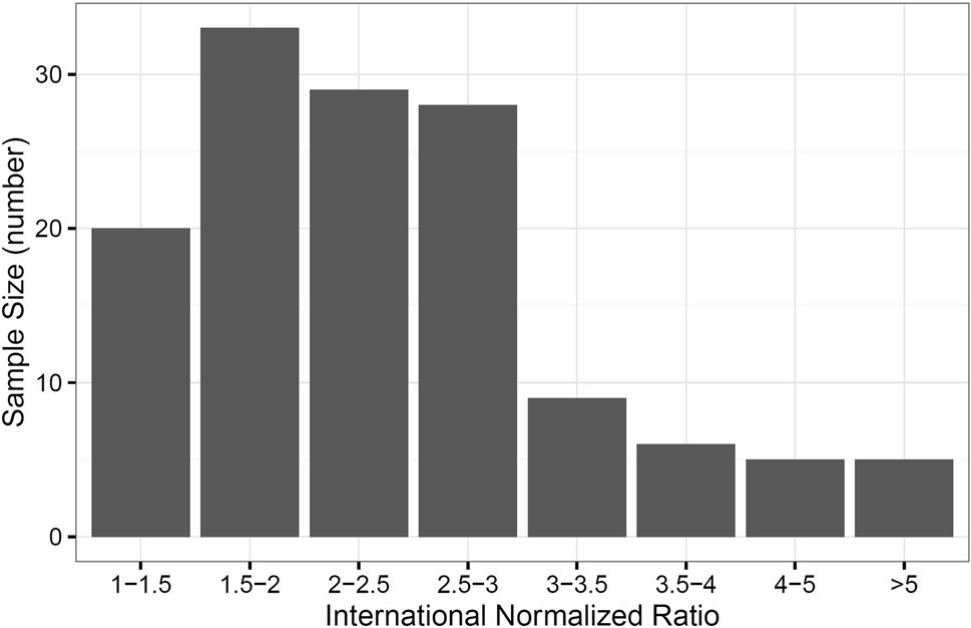
The graph shows the distribution of admission international normalized ratio where available for the cohort of patients taking warfarin at the time of admission for hip fracture.

This retrospective study is reported in accordance with the STROBE statement for cohort studies. Data for 2036 consecutive patients admitted to a tertiary referral center with fragility fracture of the femoral neck between July 2009 and July 2014 were extracted from the National Hip Fracture Database. Fifty-seven patients received no surgical treatment and were excluded from analysis. One hundred fifty-two of 1979 surgically treated patients (8%) were receiving warfarin therapy at the time of admission (Fig. 1). Internally verified data were collected on the sex, age, abbreviated mental test score (AMTS), premorbid work ability index (WAI), American Society of Anesthesiologists (ASA) grade, fracture type (intracapsular-undisplaced, intracapsular-displaced, intertrochanteric, subtrochanteric, other), operation type (internal fixation, hemiarthroplasty, or THA), and time to surgery (Table 1). Warfarin treatment status and admission INR were collected from digital patient records and laboratory reporting systems manually. Time to surgery was analyzed as a continuous variable and with specific thresholds to evaluate the potential association between warfarin treatment and time to surgery, including the likelihood of having surgery before the UK’s Best Practice Tariff (< 36 hours) [13] and achieving surgery before a known prognostic time suggested by others as important (< 48 hours) [3, 14, 21, 22, 28]. Finally, subgroup analysis was performed on patients with an INR above and below 2.5 to assess the association between INR value and outcome. A total of 135 patients were included (Fig. 2). Median INR was 2.3 (interquartile range [IQR], 2.3–9.0). Of these, 61 patients presented with an INR less than 2.5, with 74 patients presenting with an INR greater than this value. Equivalent analyses were performed to the whole cohort of patients included in this study. Discrete groupings were used instead of INR as a quantitative variable attributable to the limited sample size (n = 135). Followup analyses were performed to July 2015.

**Table 1:** Sample description for patients taking and not taking warfarin

Measure	Taking warfarin	Not taking warfarin	Total
Number of patients (%)	152 (8)	1827 (92)	1979 (100)
Age, years (median, IQR)	84 (80–88)	85 (79–90)	85 (79–90)
Male, number (%)	56 (37)	525 (29)	581 (29)
AMTS, median (IQR)	8 (6–10)	8 (4–10)	8 (5–10)
Fracture type, number (%)			
Intracapsular–undisplaced	15 (10)	164 (9)	179 (9)
Intracapsular–displaced	82 (54)	883 (48)	965 (49)
Intertrochanteric	45 (30)	662 (36)	707 (36)
Subtrochanteric	10 (7)	114 (6)	124 (6)
Other	0 (0)	4 (0)	4 (0)
ASA grade*, number (%)			
5	1 (1)	0 (0)	1 (0)
4	16 (11)	170 (9)	186 (9)
3	91 (60)	932 (51)	1023 (52)
2	38 (25)	651 (36)	689 (35)
1	3 (2)	56 (3)	59 (3)
Operation type, number (%)			
Femoral screw	72 (47)	921 (50)	993 (50)
Hemiarthroplasty	79 (52)	840 (46)	919 (46)
THA	1 (1)	66 (4)	67 (3)
Time to surgery			
Median hours (IQR)	46 (29–66)	24 (19–40)	25 (19–41)
< 36 hours, number (%)	46 (30)	1290 (71)	1336 (68)
< 48 hours, number (%)**	82 (54)	1594 (87)	1676 (85)
Length of stay, median days (IQR)	15 (12–22)	13 (9–20)	13 (9–20)
Survival, number (%)			
30-day	138 (91)	1732 (95)	1870 (95)
6-month	115 (76)	1500 (82)	1615 (82)
12-month	101 (66)	1395 (76)	1496 (76)

*American Society of Anesthesiologists (ASA) grade was missing for 21 patients; IQR = interquartile range; AMTS = Abbreviated Mental Test Score; ** includes patients operated on in < 36 hours.

### Statistical Analysis

Initially, a demographic analysis was done to present key characteristics of the cohort acquired. Variables were analyzed in a multivariable ordinary least squares regression for length of stay and the time to surgery, a logistic regression for the specific time to surgery cutoffs of less than 36 hours and less than 48 hours, and a Cox proportional hazards model for survival. Odds ratios (OR) and 95% CIs were calculated for logistic regression results and hazard ratios, and 95% CIs for the Cox proportional hazards model results. Age was modeled using restricted cubic splines. Categorical and nominal variables were modeled as factors. Significance threshold for all variables and interaction terms was set at a probability less than 0.05. All analyses were conducted using the *rms* package in R version 3.2.1 [12, 15]. Graphic production was completed using the ggplot2 package in R, version 3.2.1 [27].

The project was approved by and registered with the center’s quality improvement team. The National Hip Fracture Database is a national audit in the UK approved by the National Health Service (NHS) England Health Research Authority (HRA) Confidentiality Advisory Group.

## Results

### Time to Surgery

After controlling for sex, age, AMTS, WAI, ASA grade, fracture type, and operation type, patients taking warfarin went to surgery later than those not treated with warfarin (Table 2) (f = 86.8; p < 0.001). Results were consistent when analyzing time-interval thresholds; patients taking warfarin were less likely to go to surgery by 36 hours (OR, 0.20; 95% CI, 0.14–0.30) and less likely to go to surgery by 48 hours (OR, 0.17; 95% CI, 0.11–0.24). Median time to surgery was substantially higher in patients treated with warfarin (median, 46 hours; IQR, 29–66) compared with patients not treated (median, 24 hours; IQR, 19–40). A higher INR did not mean patients were less likely to go to surgery (Supplemental Table 1 [f = 0.3; p = 0.576]. Supplemental material is available with the online version of *CORR* ^®^).

**Table 2:** Multivariable regression results showing factors related to time to surgery

Factor	Time to surgery (hours)			< 36 hours			< 48 hours		
	df	f	p value	df	Chi square	p value	df	Chi square	p value
Sex	1	4.9	0.028	1	0.5	0.484	1	1.5	0.219
Age	4	1.6	0.161	4	7.0	0.138	4	2.0	0.741
AMTS	1	3.7	0.054	1	6.9	0.009	1	4.4	0.037
WAI	8	0.9	0.522	8	11.5	0.177	8	1.3	0.996
ASA grade	4	7.6	< 0.0001	4	10.1	0.039	4	18.6	0.001
Fracture type	4	2.3	0.059	4	2.4	0.656	4	2.9	0.575
Operation type	2	5.2	0.006	2	11.2	0.004	2	12.0	0.002
Warfarin	1	86.8	< 0.0001	1	69.9	< 0.0001	1	89.1	< 0.001

AMTS = Abbreviated Mental Test Score; WAI = Work Ability Index; ASA = American Society of Anesthesiologists.

### Length of Stay

After controlling for sex, age, AMTS, WAI, ASA score, fracture type, and operation, length of stay was found to be increased in patients treated with warfarin (Table 3) (f = 13.05; p < 0.001). Patients treated with warfarin recorded a median length of stay of 15 days (IQR, 12–22 days) compared with 13 days (IQR, 9–20 days) for those not treated with warfarin. A higher INR was not associated with greater length of stay (Supplemental Table 2 [f = 0.07; p = 0.787]. Supplemental material is available with the online version of *CORR* ^®^).

**Table 3:** Multivariable regression results showing factors related to length of stay

Factor	df	f	p value
Sex	1	23.49	< 0.001
Age	4	3.53	0.007
AMTS	1	44.32	< 0.001
ASA grade	3	12.03	< 0.001
Fracture type	4	2.84	0.023
WAI	8	1.98	0.045
Operation type	2	2.23	0.108
Warfarin	1	13.05	< 0.001

AMTS = Abbreviated Mental Test Score; ASA = American Society of Anesthesiologists; WAI = Work Ability Index.

### Survival

**Fig. 3:**
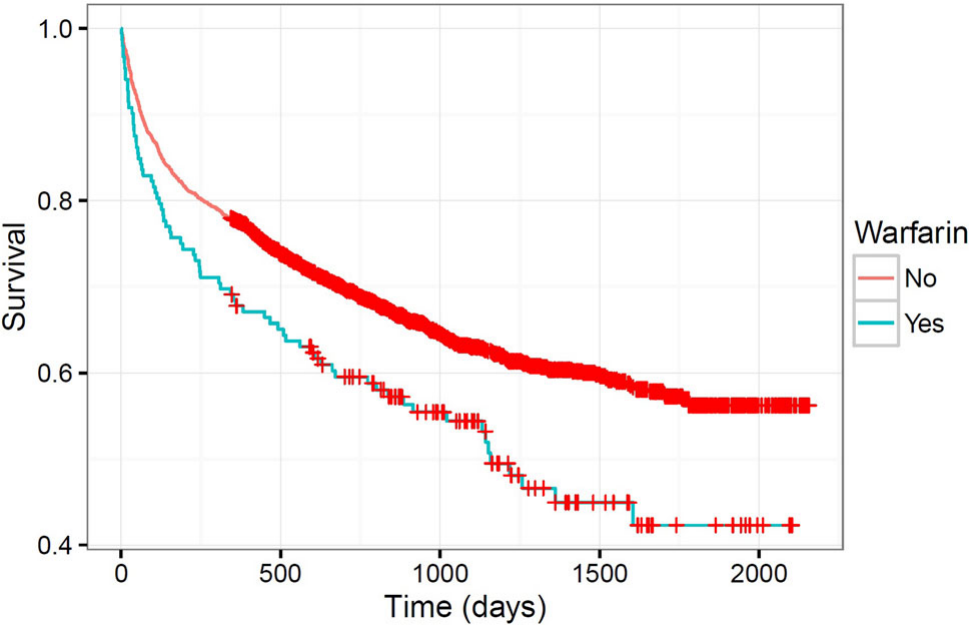
The survival curves show the effect of warfarin treatment status on survival.

After controlling for age, sex, ASA score, AMTS, WAI, fracture type, and operation, treatment with warfarin was found to be associated with reduced survival with time (chi square = 12.7; p < 0.001) (Table 4). The hazard ratio for warfarin treatment was 1.57 (95% CI, 1.21–2.04). Unadjusted survival was lower throughout followup for patients taking warfarin (chi square = 9.9; p = 0.002) (Fig. 3). A higher INR was not associated with reduced followup survival (Supplemental Table 3 [chi square = 0.1, p = 0.767]. Supplemental material is available with the online version of *CORR* ^®^).

**Table 4:** Cox proportional hazards survival model

Factor	df	Chi square	p value
Sex	1	35.0	< 0.001
Age	4	31.5	< 0.001
AMTS	1	58.0	< 0.001
WAI	8	27.9	0.001
ASA grade	4	101.5	< 0.001
Fracture type	4	12.2	0.016
Operation type	2	6.1	0.047
Time to surgery	1	0.0	0.912
Warfarin	1	12.7	< 0.001

AMTS = Abbreviated Mental Test Score; WAI = Work Ability Index; ASA = American Society of Anesthesiologists.

## Discussion

Fractures of the femoral neck are a rapidly growing international public health problem. Many of the patients who sustain this injury are elderly with multiple comorbidities, some of which require the use of warfarin therapy. The resultant coagulopathy together with the presence of these comorbidities has the potential to delay treatment and subsequently affect outcomes. We found that in our patient population, after controlling for age, sex, ASA score, AMTS, WAI, fracture type, and operation, that warfarin therapy is associated with increased time to surgery, length of stay, and decreased survival.

This study used data from the National Hip Fracture Database together with the digitally stored components of hospital patient records. Although this approach was practical for the large number of patients involved and allowed a wide range of relevant information to be gathered, it was not exhaustive in its level of detail. Some relevant information was contained only in the patient’s “paper” hospital notes, the majority of which are retained at a storage facility not in our center and with very limited access for research purposes. In particular, we were unable to control more precisely for comorbidity in our analysis. Although our study controlled for ASA grade, which has been shown to accurately reflect medical comorbidity and the likelihood of medical complications and mortality after surgery, this does not allow accurate conclusions to be drawn regarding the causes of differing outcomes between the two groups [10, 17]. Similarly, our data did not contain detailed information regarding the reasons for delays to the operating room. There are several possible difference between the two groups in this regard. The most common indication for warfarin therapy in this group of patients is atrial fibrillation (83%), followed by thromboembolic disease (18%), and mechanical heart valve (5%), with approximately 7% having multiple indications [9]. These comorbidities may cause delays in time to the operating room owing to the time needed to appropriately investigate and optimize the patient before administration of anesthesia. Similarly, the time taken to reverse the warfarin-induced coagulopathy using reversal agents in these patients may be a cause of delay. Future studies investigating the cause of these differences would need detailed analysis of drug cards and patient notes that were not possible for this study. This will be aided by the use of digital recordkeeping, which is becoming increasingly popular across the UK. Although our study involves a relatively large cohort of 1979 patients, the number taking warfarin was comparatively small. This did not allow for detailed subgroup analysis, which would have enabled us to draw more detailed conclusions regarding the relationship between admission INR and clinical outcome.

We found that after controlling for age, sex, ASA score, AMTS, WAI, fracture type, and operation type, warfarin therapy was associated with increased time to surgery. This delay has been reported in previous studies [7, 8, 16, 24], although our study is the first, to our knowledge, showing the same association after regression analysis using these prognostic factors. Ryan et al. [21] conducted a study with more than two million patients sustaining a fractured neck of the femur and reported that a time to the operating room in excess of 48 hours resulted in higher mortality, with an OR of 1.13 for the delayed group after controlling for multiple prognostic factors including comorbidity. They also showed this risk increased with time, with patients who had surgery more than 3 days after admission having a substantially increased risk of death (OR, 1.33). Our study is the most comprehensive to date to show association between warfarin therapy and delays to the operating room, and therefore should highlight the need to prevent any avoidable preoperative delays in these patients. Early warfarin reversal together with adequate investigation and optimization of these more complex patients could ensure timely, safe surgery [2, 7–9, 23, 26].

Patients receiving warfarin at the time of hospital admission for femoral neck fracture had longer lengths of hospital stay. Previous studies have come to differing conclusions regarding this matter. Ranhoff et al. [16], in a 2011 study of 1192 patients with hip fracture, found longer length of stay for warfarin users compared with nonusers. Conversely, Eardley et al. [8], in a [2014 study of 1024 patients, showed no association between length of stay and the use of warfarin at the time of admission for hip fracture. Our study differs from both of these in that we have controlled for age, sex, ASA score, AMTS, WAI, fracture type, and operation type in our analysis. Our study therefore provides the most robust evidence to date that warfarin users experience longer hospital stays after hip fracture.

We found that patients taking warfarin before their hip fractures were more likely to die during the 12 months after hip-fracture surgery. This is the first study, to our knowledge, to show poorer survival in this group of patients. This should encourage clinicians to treat warfarin therapy as a ‘red flag’ in patients sustaining a fractured neck of the femur and ensure all necessary steps are taken to ensure early surgery, involvement of all the appropriate medical teams, and appropriate levels of postoperative care.

Our study showed an association between warfarin therapy at the time of admission for hip fracture and outcome as measured by time to surgery, length of stay, and survival. Clinicians should ensure this complex group of patients receive an appropriate level of postoperative care, with early involvement of the anesthesia team. Warfarin should be recommenced in a timely manner to ensure a therapeutic INR is achieved when the patient is clinically fit for discharge. Further studies should focus on establishing the cause of these poor outcomes and identify areas for improvement in the care of these patients.

## Electronic supplementary material

Below is the link to the electronic supplementary material.

Supplementary material 1 (DOC 52 kb)
