# Supplementary material for: Do Patients Taking Warfarin Experience Delays to Theatre, Longer Hospital Stay, and Poorer Survival After Hip Fracture?
Source: Clin Orthop Relat Res. 2016 Sep 1;475(1):273–9. doi: 10.1007/s11999-016-5056-0 (PMC5174047; doi:10.1007/s11999-016-5056-0)
Supplement: Supplementary file 1 — Supplementary material 1 (DOC 52 kb) [file 11999_2016_5056_MOESM1_ESM.doc]

**Supplemental Table 1**. Multivariable regression results showing factors related to time to surgery

| Factor | Time to surgery/hours | | | < 36 hours | | | < 48 hours | | |
| --- | --- | --- | --- | --- | --- | --- | --- | --- | --- |
|  | df | f | p Value | df | Chi square | p Value | df | Chi square | p Value |
| Sex | 1 | 2.5 | 0.116 | 1 | 2.8 | 0.092 | 1 | 1.3 | 0.259 |
| Age | 4 | 0.5 | 0.736 | 4 | 2.8 | 0.589 | 4 | 5.3 | 0.255 |
| AMTS | 1 | 4.7 | 0.033 | 1 | 6.4 | 0.012 | 1 | 5.2 | 0.023 |
| WAI | 4 | 1.6 | 0.183 | 4 | 3.2 | 0.521 | 4 | 5.7 | 0.219 |
| ASA grade | 4 | 1.9 | 0.126 | 4 | 1.6 | 0.817 | 4 | 4.8 | 0.307 |
| Fracture type | 3 | 0.4 | 0.787 | 3 | 1.9 | 0.584 | 3 | 3.7 | 0.292 |
| Operation type | 2 | 0.6 | 0.573 | 2 | 0.0 | 0.993 | 2 | 1.3 | 0.512 |
| INR > 2.5 | 1 | 0.3 | 0.576 | 1 | 1.5 | 0.228 | 1 | 0.4 | 0.522 |
|  |  |  |  |  |  |  |  |  |  |

**Supplemental Table 2**. Multivariable regression results showing factors related to length of stay

| Factor | df | f | p Value |
| --- | --- | --- | --- |
| Sex | 1 | 1.76 | 0.1883 |
| Age | 4 | 0.8 | 0.5305 |
| AMTS | 1 | 6.08 | 0.0155 |
| WAI | 4 | 1.06 | 0.3831 |
| ASA grade | 3 | 0.88 | 0.4557 |
| Fracture type | 3 | 0.09 | 0.9643 |
| Operation type | 2 | 0.1 | 0.9045 |
| INR > 2.5 | 1 | 0.07 | 0.7868 |

**Supplemental Table 3**. Cox proportional hazards survival model

| Factor | df | Chi square | p Value |
| --- | --- | --- | --- |
| Sex | 1 | 0.2 | 0.645 |
| Age | 4 | 2.7 | 0.607 |
| AMTS | 1 | 7.1 | 0.008 |
| WAI | 4 | 8.9 | 0.063 |
| ASA grade | 4 | 21.7 | 0.000 |
| Fracture type | 3 | 2.1 | 0.545 |
| Operation type | 2 | 0.6 | 0.753 |
| Time to surgery | 1 | 0.0 | 0.846 |
| INR > 2.5 | 1 | 0.1 | 0.767 |

The results show the model output for the subgroup analysis comparing two groups of patients with warfarin treatment with an international normalized ratio (INR) below and above 2.5 respectively; AMTS = Abbreviated Mental Test Score; WAI = work ability index; ASA = American Society of Anesthesiologists.
